# Association of trial registration with the results and conclusions of published trials of new oncology drugs

**DOI:** 10.1186/1745-6215-10-116

**Published:** 2009-12-16

**Authors:** Nicolas Rasmussen, Kirby Lee, Lisa Bero

**Affiliations:** 1 History & Philosophy of Science, University of New South Wales, Sydney 2052, Australia; 2 Department of Clinical Pharmacy, University of California, 3333 California St., San Francisco, CA 94118, USA

## Abstract

**Background:**

Registration of clinical trials has been introduced largely to reduce bias toward statistically significant results in the trial literature. Doubts remain about whether advance registration alone is an adequate measure to reduce selective publication, selective outcome reporting, and biased design. One of the first areas of medicine in which registration was widely adopted was oncology, although the bulk of registered oncology trials remain unpublished. The net influence of registration on the literature remains untested. This study compares the prevalence of favorable results and conclusions among published reports of registered and unregistered randomized controlled trials of new oncology drugs.

**Methods:**

We conducted a cross-sectional study of published original research articles reporting clinical trials evaluating the efficacy of drugs newly approved for antimalignancy indications by the United States Food and Drug Administration (FDA) from 2000 through 2005. Drugs receiving first-time approval for indications in oncology were identified using the FDA web site and Thomson Centerwatch. Relevant trial reports were identified using PubMed and the Cochrane Library. Evidence of advance trial registration was obtained by a search of clinicaltrials.gov, WHO, ISRCTN, NCI-PDQ trial databases and corporate trial registries, as well as articles themselves. Data on blinding, results for primary outcomes, and author conclusions were extracted independently by two coders. Univariate and multivariate logistic regression identified associations between favorable results and conclusions and independent variables including advance registration, study design characteristics, and industry sponsorship.

**Results:**

Of 137 original research reports from 115 distinct randomized trials assessing 25 newly approved drugs for treating cancer, the 54 publications describing data from trials registered prior to publication were as likely to report statistically significant efficacy results and reach conclusions favoring the test drug (for results, OR = 1.77; 95% CI = 0.87 to 3.61) as reports of trials not registered in advance. In multivariate analysis, reports of prior registered trials were again as likely to favor the test drug (OR = 1.29; 95% CI = 0.54 to 3.08); large sample sizes and surrogate outcome measures were statistically significant predictors of favorable efficacy results at p < 0.05. Subgroup analysis of the main reports from each trial (n = 115) similarly indicated that registered trials were as likely to report results favoring the test drug as trials not registered in advance (OR = 1.11; 95% CI = 0.44 to 2.80), and also that large trials and trials with nonstringent blinding were significantly more likely to report results favoring the test drug.

**Conclusions:**

Trial registration alone, without a requirement for full reporting of research results, does not appear to reduce a bias toward results and conclusions favoring new drugs in the clinical trials literature. Our findings support the inclusion of full results reporting in trial registers, as well as protocols to allow assessment of whether results have been completely reported.

## Background

The clinical trial literature is known to be a biased source of evidence on therapeutic efficacy. A number of potential mechanisms accounting for bias have been identified [1]. Selective publication of "statistically significant" results favoring new treatments is one well-established problem [2-7]. Intrinsic factors of trial design can also lead to bias in randomized controlled trials of drug efficacy [8,9]. Specific design features that have been associated with bias in reported trials include blinding[8,10,11], sample size [9,11-13], and choice of comparator [14-17]. Particular data handling and analysis procedures have been associated with bias [13,18,19] and also incomplete reporting of outcomes [2,20-22]. Trials and investigators supported by pharmaceutical companies are more likely to report results and conclusions favorable towards the sponsor's product compared to placebo [13,14,23,24], or to the drugs of other manufacturers [11,25], through mechanisms that remain incompletely characterized.

The public registration of clinical trials has been introduced, partly to satisfy ethical obligations of human experimentation [26-30], and partly to reduce bias via several mechanisms (increasing scrutiny of design, encouraging publication, and encouraging full outcome reporting) [7,31-33]. United States Federal law in 1997 mandated a national registry http://www.clinicaltrials.gov, which was operational by 1999 [30]. However the registry was at first little used except in a few fields, notably oncology (where it facilitated patient recruitment) [34]. Since the International Committee of Medical Journal Editors instituted a policy requiring prior trial registration for publication in cooperating journals from late 2005 [29], registration has become much more common and may soon be a universal regulatory requirement [30,35]. However, doubts remain about registration's adequacy in principle for preventing bias due to trial design [36,37]. Furthermore, there is evidence that registration alone may not greatly influence biased outcome reporting: among registered trials in cardiology, rheumatology, and gastroenterology that have been published, many showed discrepancies between primary outcomes as reported and outcomes as registered, the vast majority of which tended to favor statistically significant results [37]. Similarly, advance registration of trials may not greatly discourage selective publication: the majority of registered trials across all fields remain unpublished, and are less likely to be published if industry sponsored [38].

One of the main goals of trial registration is to reduce bias in the literature through some combination of effects on the mechanisms favoring new treatments. Oncology is one field in which published results and conclusions have been found to exhibit a bias favoring new drugs, associated statistically with drugmaker sponsorship and with particular trial design features [39]. A minority of oncology trials registered before September 2004 have reached publication, indicating that the possibility of publication bias in this field also remains [40]. In oncology, high quality randomized clinical trials (those of the National Cancer Institute's Cooperative Oncology Groups) have in the long run favored new treatments very nearly as often as comparator treatments, making this field unusually advantageous for studying predictors of favorable outcomes in that conditions approximately consistent with equipoise have been demonstrated [41]. To assess whether registration is associated with any net difference in the prevalence of results and conclusions favoring new drugs in the clinical trial literature, we conducted a cross-sectional study of randomized clinical trial reports of oncology drugs approved by the United States Food and Drug Administration (FDA) from 2000 through 2005. We hypothesized that advance trial registration is associated with statistically non-significant efficacy outcomes in published trial reports, when controlling for other known predictors of reported results such as funding source, publication characteristics, and trial design characteristics.

## Methods

### Search strategy

We searched drug approvals by year on the FDA web site http://www.accessdata.fda.gov/scripts/cder/drugsatfda/index.cfm as well as Thomson CenterWatch http://www.centerwatch.com/drug-information/fda-approvals/. We identified 25 relevant drugs granted first-time FDA approval for oncology indications in the 2000-2005 period, including both new molecular entities and older drugs receiving new indications. We electronically searched PubMed and the *Cochrane Library *in mid-October 2007 to identify reports of randomized controlled trials including these drugs. Search terms were the generic name(s) of the drugs of interest, in PubMed limited to "randomized" or "randomised", "clinical trials", and "humans". Our search included articles published in any language. The search was updated in late February 2009 to expand the sample so as to include reports published through the end of 2008. Thus trial reports on all drugs of interest were collected for a full nine-year interval surrounding FDA approval, except for those drugs approved in 2005, for which only eight years of literature was searched.

### Published Articles

We reviewed abstracts of all citations and retrieved articles based on the following inclusion criteria: (1) prospective randomized controlled trial; (2) oncology drug of interest compared to a treatment arm not including that drug (e.g., placebo or active control); (3) efficacy measured in human cancer patients; (4) efficacy measured in terms of direct effects on malignancy (not treatment-related pathology or supportive care only); (5) original research, defined as studies that appeared to present original data and did not specifically state that they were reviews. Studies with the primary objective of assessing the effect of the oncology drug of interest combined with another treatment were included, so long as one treatment arm including the drug of interest was compared with an otherwise identical arm lacking it, or with another treatment in its place. In the case of oxaliplatin, approved in 2002 only in combination with 5-fluorouracil and leucovorin, oxaliplatin was defined as the chief drug of interest and only trials comparing the approved combination to a treatment arm not containing oxaliplatin were included.

The following exclusion criteria were used to screen all abstracts: (1) studies not evaluating clinical efficacy outcomes (e.g., pharmacodynamic studies); (2) editorials, letters-to-the-editor, commentaries, abstracts, reviews, studies only describing a trial but not results; (3) studies presenting only an economic analysis; (4) retrospective analysis of outcome predictors not reporting any new efficacy data; (5) studies in which the oncology drug of interest was present in all arms of the trial; (6) studies published more than four years before or after the year of the oncology drug's approval in the 2000 to 2005 interval; (7) studies presenting pooled data from multiple trials, where individual trial results are not interpretable separately; (8) studies in which the primary efficacy outcome is not assessed statistically or not reported sufficiently for statistical assessment; and (9) strictly biological or *in vitro *analyses. We did not identify any exact duplicate publications. As we were interested in published trial reports, we did include multiple publications from the same underlying trial if the publications reported different data. Where any doubt about a publication's eligibility for inclusion remained after reading the abstract, it was provisionally included for a full reading.

### Data extraction

One investigator (N.R.) who was not blinded to author names and affiliations, funding sources, and financial disclosure extracted all data from each article. All data were extracted from articles before a search of the registration status of the underlying trial was conducted. A second coder (K.L.) who was blinded to registration status (except where it was stated in the publication) independently extracted data on blinding, identification and results of primary outcomes, and author conclusions. Inter-rater agreement was very good to excellent, with Kappa values ranging from 0.85 to 0.95 for double-coded items. In cases of disagreement the two coders discussed the papers and reached agreement.

We extracted data on trial registration status and publication characteristics that have been shown to be independently related to publication of favorable results or conclusions of drug studies, as follows [8-12,23].

### Registration status

For all articles the registration status of the underlying trial was sought by searching cancer type and drug names in key databases. These included the U.S. National Institutes of Health trial registry http://www.clinicaltrials.gov, the International Standard Randomized Controlled Trial Number Register http://isrctn.org, the WHO International Clinical Trials Registry platform http://www.who.int/trialsearch/, the U.S. National Cancer Institute PDQ Comprehensive Cancer database http://www.cancer.gov/Search/SearchClinicalTrialsAdvanced.aspx, and where available, the corporate trial registries and databases of the manufacturers of the drugs. Where the underlying trial could unambiguously be identified in a registry or registries (by therapy protocols, inclusion criteria, patient numbers, location, commencement date, etc.), the earliest recorded date and place of registration was noted. For analysis, only reports of trials registered in a year prior to the earliest year of publication of results were classified as registered. Some registered trial reports did not meet this condition, as registration became a precondition of publication in many journals during the interval studied and post-hoc registration was allowed to satisfy this requirement. Sixteen of the 83 papers coded as unregistered appeared to have been registered in the same year as the first report, thus presumably after trial completion.

### Publication characteristics

#### Peer Review Status

Each article was classified as peer reviewed or non-peer reviewed based on information found on the website of the journal where the article was published. A publication was considered peer reviewed if the website mentioned that the journal had a peer review process or if it was stated that the manuscripts were evaluated by at least one external expert in the field; otherwise, a publication was considered non-peer reviewed.

#### Impact Factor

For each article, impact factor for the journal in the year of publication was obtained from the Institute for Scientific Information [42].

#### Main report

Trial reports were classified as 1) first or main reports, if they were the earliest article in a given drug's publication sampling interval reporting results on a particular trial's primary efficacy outcome measure in the full patient population, or 2) subsidiary reports if an earlier publication in the sampling interval had disclosed results of the primary outcome measure.

### Study design characteristics

#### Comparison group

The comparator for the primary outcome was classified as 1) placebo controlled, or 2) drug versus other treatment only. In drug versus other treatment comparisons involving two different oncology drugs of interest, the "test" drug was defined as the newer drug (most recent FDA approval date) and the older drug as the "comparator".

If a trial included multiple treatment arms, the placebo arm was treated as the comparator arm if present. If trials included multiple arms differing in dosage of the oncology drug of interest, the arm specifying the higher cumulative dosage was treated as the "test" arm whenever possible. If a publication reported results from multiple trials, the trial with the largest number of patients randomized was coded.

#### Equivalency or Superiority trial

Articles were classified as 1) equivalency/noninferiority trial if stated in the publication or 2) superiority trial.

#### Type of primary outcome measures

The preferred efficacy outcome measure recommended by the FDA for trials of antimalignancy therapies in this period was overall survival [43]. We classified the primary efficacy outcome reported in a publication as 1) overall survival or an equivalent measure (e.g. time to death) or 2) surrogate, if a surrogate measure was specified as the primary outcome. If no primary efficacy outcome was stated, or more than one "primary" outcome, the most emphasized efficacy outcome in the publication was designated the main outcome and treated as primary. In such cases we operationalized "most emphasized" according to word count in the results section, or if word count was about equal, the outcome discussed first. Identification of primary outcome measure was double-coded with high agreement (although kappa cannot be calculated because the number of possible outcome measures is indefinite).

#### Blinding

Blinding of treatment group allocation was classified on a scale of 0 to 3 based on the scheme of Chalmers et al. [44], with one point each for procedures assuring that patients were unaware of their allocation, that treating physicians and other patient care personnel were unaware, and that those evaluating the primary efficacy endpoint were unaware. If papers stated only that they were "double blind" without specifying procedures, they were classified with only one point. For analysis, we dichotomized blinding into "stringent" (score of 2 or 3) or "nonstringent" (score of 0 or 1).

#### Sample Size

We recorded the total number of patients randomized to all treatments as described in the article, or total number enrolled if number randomized was not stated.

### Statistical Significance of Primary Outcome

For each report, the results described for the primary efficacy outcome were categorised as 1) favorable if the result was statistically significant (p < 0.05 unless more stringent criteria were specified) and in the direction of the test drug being more efficacious, or 2) inconclusive if the result did not reach statistical significance, or 3) unfavorable if the result was statistically significant in the direction of the comparator treatment being more efficacious. If a paper explicitly stated that it was designed as a non-inferiority or equivalency study and no significant efficacy difference between the two comparison treatments was reported, the result was coded as favorable. For analysis, conclusions were dichotomised as favorable or not (combining inconclusive and unfavorable).

### Conclusion

The overall conclusions reached in the published reports were categorised as 1) favorable, if the test drug was preferred to comparator, 2) equivalent if the test drug was described as about equal to comparator, or 3) unfavorable, if the comparator was preferred to the test drug. If an article did not clearly state that one of the treatments was better or if the comparison treatments had different but balanced advantages, the conclusion was coded as "test drug equivalent to comparator". If a study explicitly stated that it was designed as a non-inferiority or equivalency trial and the comparison treatments were said to be about equal, the conclusion was coded as favorable. For analysis, conclusions were dichotomized as favorable or not (combining equivalent and unfavorable).

### Sponsorship information

#### Funding source

The funding source(s) of the published reports were categorized as 1) industry, 2) private non-profit, 3) government, 4) other, 5) no funding, 6) none disclosed.

#### Financial ties

Data about the financial ties of each author were extracted and coded for 1) whether or not there were ties of employment, research funding, consulting, stock ownership, or honoraria disclosed with the sponsor of the study (yes, no, or none disclosed), and 2) whether or not there were these same financial ties disclosed with any other company (yes, no, or none disclosed).

For analysis these categories were collapsed into two classes of sponsorship status, 1) sponsored by the test drug maker, if such a funding source for the trial was acknowledged explicitly, or where any author listed a relevant corporate address or employment, and 2) not commercially sponsored, if neither of the preceding categories applied. Supply of materials, or consulting or honoraria (etc.) on the part of authors did not in themselves qualify as sponsorship. Only one study was sponsored by the manufacturer of the comparator [45], and for analysis the paper was recoded as if the comparator (in fact an even newer drug) were the test drug.

### Analysis

We report frequencies of the different characteristics of each article by registration status of the trial. Proportions of reports with favorable results or conclusions were first analyzed using univariate logistic regression to identify associations between independent variables and favorable results and conclusions. Although impact factor and sample size were continuous variables, they were modeled categorically by quartile because their effects were clearly non-linear. An exploration of interactions between trial and publication characteristics revealed no interactions except between trial sample size and journal impact factor (see Results, below).

To control for multiple variables simultaneously, we carried out multivariate logistic regression analysis and calculated odds ratios. These models included registration status as our primary predictor and all factors statistically associated either in the literature or in our univariate models with favorable results or conclusions. The natural log of sample size was treated as a numeric predictor. For our primary analysis, we conducted the multivariate regression on our full sample (n = 137). We carried out pre-planned subgroup analyses of industry-sponsored studies only (n = 109), and first or main reports only (i.e., one report per trial, n = 115). We also assessed sensitivity of our results to our definition of registration by repeating the primary multivariate analysis with both less stringent definitions of advance trial registration (any evidence of registration) and more stringent (evidence of registration in a year at least two years prior to publication). Data were analyzed with SAS software (version 9.1, SAS Institute, Cary, NC, USA).

## Results

**Figure 1:**
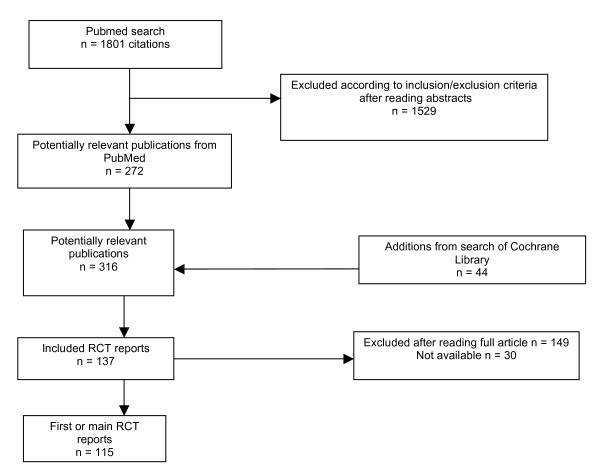
**Identification of trial reports for inclusion**.

As designed this study required a sample of 116 included reports to achieve 80% power, assuming that 50% of reports from registered trials would describe favorable results and 75% from unregistered trials, and equal numbers of the two categories. Our actual full sample consisted of 137 trial reports meeting inclusion criteria (see Figure 1), and provided 85% power to distinguish the expected proportions. The sample characteristics are shown in Table 1. Thirty-nine percent (54/137) described trials registered prior to the year of the first publication. The majority of both registered and unregistered trial reports were published in peer reviewed journals, did not describe stringent blinding, reported on a surrogate primary efficacy outcome measure, and were sponsored by the test drug maker. Fifty-eight percent (80/137) of the trial reports described results that were statistically significant in favor of the test drug for the primary efficacy outcome. Seventy-two percent (98/137) had conclusions that favored the test drug.

**Table 1:** Characteristics of included articles by registration status (n = 137)

Characteristic	Prior registration N = 54	No Prior Registration N = 83
Publication characteristics		

Peer reviewed	53 (98%)	76 (92%)

Year of publication		
1996-2002	0 (0%)	29 (35%)
2003-2004	12 (22%)	28 (34%)
2005-2006	14 (26%)	19 (23%)
2007-2008	28 (52%)	7 (8%)

Impact factor, median (interquartile range)*	15.5 (10.9-34.8)	5.8 (4.0-10.5)

First or main report	45 (83%)	70 (84%)

Study design characteristics		

Comparison group		

Placebo	40 (74%)	32 (39%)

Active treatment only	14 (26%)	51 (62%)

Non-inferiority trial - Yes	2 (4%)	9 (11%)

Primary efficacy outcome = survival	21 (39%)	9 (11%)

Stringent blinding (2/3 points)	16 (30%)	20 (24%)

Sample size, median (interquartile range)	696 (150-923)	283 (99-565)

Statistical significance of primary outcome		

Favorable to test drug	36 (67%)	44 (53%)

Inconclusive	18 (33%)	37 (45%)

Unfavorable	0 (%)	2 (2%)

Conclusion		

Favorable to test drug	43 (80%)	55 (66%)

About equal	10 (18%)	22 (27%)

Unfavorable	1 (2%)	6 (7%)

Sponsored by test drug maker	47 (87%)	62 (75%)

*Only 127 articles were published in journals with a corresponding impact factor.

Table 2 shows the results of univariate analysis of the full sample. Reports of trials with large sample sizes were significantly more likely to describe results and conclusions favoring the test drug, as were reports published in higher impact journals. Reports of trials sponsored by the maker of a test drug were significantly more likely to reach conclusions favoring the drug than non-sponsored trial reports. We found no statistically significant difference in results or conclusions between registered and non-registered trial reports. Published reports of registered trials were as likely to describe primary outcome results (OR = 1.77; 95% CI = 0.87 to 3.61) and conclusions (OR = 1.99; 95% CI = 0.89 to 4.45) favoring the test drug compared to reports of non-registered trials.

**Table 2:** Association between characteristics of articles and statistically significant results or conclusions that favor the test drug: Univariate logistic regression (n = 137).

		Results Favor Test Drug			Conclusions Favor Test Drug		
**Characteristic**	**Category**	**Favorable n/Total n (%)**	**OR** **(95% CI)**	***P***	**Favorable n/Total n (%)**	**OR** **(95% CI)**	***P***

Trial registration before publication	No	44/83 (53)	1.00		55/83 (66)	1.00	

	Yes	36/54 (67)	1.77 (0.87-3.61)	0.115	43/54 (80)	1.99 (0.89-4.45)	0.093

Year of publication	1996-2002	16/29 (55)	1.00		21/29 (72)	1.00	

	2003-2004	27/40 (68)	1.69 (0.63-4.53)	0.299	30/40 (75)	1.14 (0.39-3.38)	0.809

	2005-2006	18/33 (55)	0.98 (0.36-2.66)	0.961	20/33 (61)	0.59 (0.20-1.71)	0.328

	2007-2008	19/35 (54)	0.97 (0.36-2.59)	0.943	27/35 (77)	1.29 (0.41-4.00)	0.664

Impact Factor*	Quartile 1 (1.55-4.44)	15/32 (47)	1.00		19/32 (59)	1.00	

	Quartile 2 (4.45-10.44)	19/33 (58)	1.54 (0.58-4.10)	0.389	22/33 (67)	1.37 (0.50-3.76)	0.543

	Quartile 3 (10.45-17.15)	20/35 (57)	1.51 (0.58-3.96)	0.402	26/35(74)	1.98 (0.70-5.57)	0.197

	Quartile 4 (17.16-52.59)	21/27 (78)	3.97 (1.27-12.43)	0.018	24/27 (89)	5.47 (1.36-22.01)	0.017

Blinding	Stringent	18/36 (50)	1.00		25/36 (69)	1.00	

	Not stringent	62/101 (61)	1.59 (0.74-3.42)	0.236	73/101 (72)	1.15 (0.50-2.64)	0.747

Sample Size	Quartile 1 (6-122)	10/34 (29)	1.00		19/34 (56)	1.00	

	Quartile 2 (123-352)	22/34 (65)	4.40 (1.59-12.19)	0.004	24/34 (71)	1.90 (0.70-5.16)	0.211

	Quartile 3 (353-772)	22/35 (63)	4.06 (1.48-11.12)	0.006	26/35 (74)	2.28 (0.83-6.30)	0.112

	Quartile 4 (773-8010)	26/34 (76)	7.8 (2.64-23.03)	<0.001	29/34 (85)	4.58 (1.43-14.69)	0.011

Primary efficacy outcome	Survival	17/30 (57)	1.00		21/30 (70)	1.00	

	Surrogate	63/107 (59)	1.10 (0.48-2.48)	0.828	77/107 (72)	1.10 (0.45-2.67)	0.833

Comparison group	Active comparator	35/65 (54)	1.00		45/65 (69)	1.00	

	Placebo	45/72 (63)	0.70 (0.35-1.39)	0.306	53/72 (74)	0.81 (0.38-1.70)	0.571

Sponsored	No	14/28 (50)	1.00		15/28 (54)	1.00	

	Yes	66/109 (61)	1.54 (0.67-3.54)	0.314	83/109 (76)	2.77 (1.17-6.56)	0.021

*Only 127 articles were published in journals with a corresponding impact factor.

In addition to registration status, we included industry sponsorship and sample size in our multivariate analysis because of their statistical significance in the univariate analysis. We also included stringency of blinding, outcome measure type, and comparator treatment type as these trial characteristics had been associated with direction of results in similar studies [9,11,13,46]. Journal impact factor was dropped from multivariate analysis because interaction testing found that its correlation with favorable results was accounted for by sample size. There was no interaction between stringency of blinding and surrogate outcome measure, or among any other variables. In the multivariate analysis (Table 3), reports of registered trials were again as likely to describe results favoring the test drug (OR = 1.29; 95% CI = 0.54 to 3.08), and to reach favorable conclusions (OR = 1.56; 95% CI = 0.60 to 4.05). Reports of trials with surrogate outcome measures and large sample sizes (natural log) were significantly more likely to describe results favoring the test drug (p = 0.027 and p < 0.001 respectively). Large sample size was a significant predictor of conclusions favoring the test drug (p = 0.006) in the multivariate analysis.

**Table 3:** Association between characteristics of articles and statistically significant outcome or conclusions that favor the test drug: Multivariate logistic regression (full sample, n = 137)

		Results Favor Test Drug			Conclusions Favor Test Drug		
**Characteristic**	**Category**	**Favorable n/Total n (%)**	**OR** **(95% CI)**	***P***	**Favorable n/Total n (%)**	**OR** **(95% CI)**	***P***

Trial registration before publication	No	44/83 (53)	1.00		55/83 (66)	1.00	

	Yes	36/54 (67)	1.29 (0.54-3.08)	0.566	43/54 (80)	1.56 (0.60-4.05)	0.363

							

Blinding	Stringent	18/36 (50)	1.00		25/36 (69)	1.00	

	Not stringent	62/101 (61)	2.33 (0.95-5.76)	0.066	73/101 (72)	1.51 (0.59-3.91)	0.394

							

Sample Size	Natural log	-	2.28 (0.87-4.90)	<0.001	-	1.77 (1.18-2.66)	0.006

							

Comparison group	Active comparator	35/65 (54)	1.00		45/65 (69)	1.00	

	Placebo	45/72 (63)	2.06 (0.87-4.90)	0.101	53/72 (74)	1.47 (0.59-3.63)	0.410

							

Primary efficacy outcome	Survival	17/30 (57)	1.00		21/30 (70)	1.00	

	Surrogate	63/107 (59)	3.42 (1.15-10.14)	0.027	77/107 (72)	3.04 (0.95-9.68)	0.061

							

Sponsored	No	14/28 (50)	1.00		15/28 (54)	1.00	

	Yes	66/109 (61)	1.01 (0.37-2.67)	0.999	83/109 (76)	2.01 (0.76-5.31)	0.157

For the subset of trial reports sponsored by the test drug makers (n = 109), results were essentially the same as for the full set, reports of registered trials being as likely to describe favorable results (OR = 1.37; 95% CI = 0.54 to 3.44). We also conducted a multivariate analysis for the subgroup of first or main reports (n = 115), including only one report per underlying trial (Table 4). As with the full set of publications, we did not find a statistically significant difference between registered and non-registered trials in the reporting of favorable results or conclusions. Published registered trials were as likely to report results favoring the test drug (OR = 1.11; 95% CI = 0.44 to 2.80). Results only differed from the full sample multivariate analysis in that published trials with nonstringent blinding were significantly more likely to report results favoring the test drug (p = 0.028), whereas surrogate outcome measure no longer reached significance as a predictor of results, indicating that this latter trial characteristic was over-represented among multiply-published trials.

**Table 4:** Association between characteristics of trials and statistically significant outcome or conclusions favoring the test drug: Multivariate logistic regression (first/main reports only, n = 115)

		Results Favor Test Drug			Conclusions Favor Test Drug		
**Characteristic**	**Category**	**Favorable n/Total n (%)**	**OR** **(95% CI)**	***P***	**Favorable n/Total n (%)**	**OR** **(95% CI)**	***P***

Trial registration before publication	No	34/70 (49)	1.00		46/70 (66)	1.00	

	Yes	28/45 (62)	1.11 (0.44-2.80)	0.832	34/45 (76)	1.10 (0.41-2.98)	0.854

							

Blinding	Stringent	13/31 (42)	1.00		20/31 (65)	1.00	

	Not stringent	49/84 (58)	3.03 (1.13-8.16)	0.028	60/84 (71)	1.86 (0.69-5.05)	0.221

							

Sample size	Natural log	-	2.20 (1.40-3.45)	<0.001	-	1.65 (1.09-2.51)	0.019

							

Comparison group	Active comparator	24/51 (47)	1.00		33/51 (65)	1.00	

	Placebo	38/64 (59)	2.33 (0.89-6.07)	0.084	47/64 (73)	1.98 (0.73-5.41)	0.182

							

Primary efficacy outcome	Survival	14/25 (56)	1.00		17/25 (68)	1.00	

	Surrogate	48/90 (53)	2.76 (0.82-9.28)	0.102	63/90 (70)	2.86 (0.80-10.18)	0.105

							

Sponsored	No	13/27 (48)	1.00		15/27 (56)	1.00	

	Yes	49/88 (56)	1.02 (0.37-2.84)	0.965	65/88 (74)	1.94 (0.71-5.29)	0.194

A sensitivity analysis of our definition of prior registration yielded qualitatively similar results for the different definitions. The more stringent definition of prior registration (two years or more before first publication year) in our multivariate model indicated that reports of registered trials were at least as likely to describe favorable results (OR = 1.58; 95% CI = 0.65 to 3.86) as compared with our basic definition. The less stringent definition (any evidence of registration) had an effect in the opposite direction (OR = 0.72; 95% CI = 0.30 to 1.72), suggesting that the timing of trial registration may be a variable worthy of further study.

## Discussion

We examined the association of trial registration and the results and conclusions of published randomized controlled trials of new oncology drugs. We found, contrary to expectations, that published reports of pre-registered trials were as likely to describe results favoring the test drug as non-registered trials, even when controlling for other important predictors of statistical significance of results, including trial sample size, blinding, and industry sponsorship. Reports of registered trials in our sample actually appear more likely to favor new drugs (though not significantly); if so, one possible explanation is that trials expected to reflect favorably on new drugs were registered early.

Our findings argue for measures in addition to trial registration alone to ensure that statistically non-significant results are fully reported. Public access to statistically non-significant results is essential for conducting valid meta-analysis, advancing science, and enabling clinical decisions based on complete evidence [47-49]. Issues about the quality, assessment and presentation of non-peer-reviewed data in trial registers must be resolved, along with commercial concerns, without undue delay [30,48]. However, given the evidence that particular trial design features were associated with reported results, notwithstanding registration, our findings also suggest that results access through registries alone may never entirely eliminate the bias favoring new drugs in the clinical trial literature.

It might be argued that many registered "negative" trials of these recent oncology drugs have not yet been completed, or not yet submitted for publication, or that they have been rejected multiple times by journals. However, previous studies suggest that authors' decisions not to submit manuscripts, rather than journal rejection, account for the majority of unpublished studies with statistically insignificant results [2,3,50,51]

Our study has several limitations. We focused on oncology drugs because they were most likely to include a sample of registered trials. Thus, our findings may not be generalizable to other classes of drugs. We were not able to examine trials of drugs that were never approved by the FDA as this list of drugs is not provided by the FDA. Our study design did not allow us to assess trials that were not published. In addition, to focus on trials of new therapies, we excluded studies published more than four years before or after the year of the oncology drug's approval in the 2000-2005 interval. Since trials with positive results are published significantly earlier than trials with negative results (4.3 yrs vs 6.5 yrs, respectively) [4], we may have failed to identify some studies yet to be published on the more recently approved drugs. However, these latter two limitations apply equally to prior registered and non-registered trials. We examined only the primary efficacy outcomes of the trials, and thus cannot determine whether trial registration might influence the reporting of secondary outcomes or safety outcomes. Lastly, trial registration has become increasingly common. Thus, our cross-sectional study offers only an early look at the effects of trial registration on results and conclusions in a particular sample of registered and unregistered trial reports. However, subsequent studies on the association of registration with reported results will be constrained to a before/after design and its attendant shortcomings.

## Conclusions

Trial registration alone, without a requirement for full reporting of research results, does not appear to reduce a bias toward results and conclusions favoring new drugs in the clinical trials literature. Our findings support the inclusion of full results reporting in trial registers, as well as measures to allow assessment of whether results have been completely reported. However, bias related to study design may remain. Possible policy solutions for design bias include advance review of trial protocols, compliance with existing reporting requirements, and adherence to best research practices.

## Competing interests

The authors declare that they have no competing interests.

## Authors' contributions

All three authors contributed to the conception and design of the study, acquisition of data, and analysis and interpretation of data. LB and NR drafted the manuscript, and all authors provided critical revision. All authors have had full access to all of the data in the study and take responsibility for the integrity of the data and the accuracy of the data analysis.
